# Age-related sensitivity and pathological differences in infections by 2009 pandemic influenza A (H1N1) virus

**DOI:** 10.1186/1743-422X-8-52

**Published:** 2011-02-08

**Authors:** Shihui Sun, Guangyu Zhao, Wenjun Xiao, Jingya Hu, Yan Guo, Hong Yu, Xiaohong Wu, Yadi Tan, Yusen Zhou

**Affiliations:** 1 State Key Laboratory of Pathogen and Biosecurity, Beijing Institute of Microbiology and Epidemiology, Beijing 100071, China

## Abstract

**Background:**

The highly pandemic 2009 influenza A H1N1 virus infection showed distinguished skewed age distribution with majority of infection and death in children and young adults. Although previous exposure to related antigen has been proposed as an explanation, the mechanism of age protection is still unknown.

**Methods:**

In this study, murine model of different ages were inoculated intranasally with H1N1 (A/Beijing/501/09) virus and the susceptibility and pathological response to 2009 H1N1 infection were investigated.

**Results:**

Our results showed that the younger mice had higher mortality rate when infected with the same dose of virus and the lethal dose increased with age. Immunohistochemical staining of H1N1 antigens in mice lung indicated infection was in the lower respiratory tract. Most bronchial and bronchiolar epithelial cells in 4-week mice were infected while only a minor percentage of those cells in 6-month and 1-year old mice did. The young mice developed much more severe lung lesions and had higher virus load in lung than the two older groups of mice while older mice formed more inducible bronchus-associated lymphoid tissue in their lungs and more severe damage in spleen.

**Conclusions:**

These results suggest that young individuals are more sensitive to H1N1 infection and have less protective immune responses than older adults. The age factor should be considered when studying the pathogenesis and transmission of influenza virus and formulating strategies on vaccination and treatment.

## Background

In early 2009, a novel swine-origin influenza A (H1N1) virus emerged and then spread rapidly around the world [1]. WHO declared a pandemic in June 2009 when confirmed infections were reported in total of 74 countries and territories [2]. To date, the number of laboratory-confirmed death from this pandemic influenza is up to 16000 cases in more than 200 countries [3]. Compared to the 1918 strain of H1N1 virus which claimed millions of lives, this 2009 strain is moderate and the vast majority of cases were relatively mild and uncomplicated with similar symptoms as seasonal influenza and recovered in a few days [4]. The severity of pneumonia caused by the 2009 H1N1 influenza virus was intermediate between that due to seasonal H1N1 virus and highly pathogenic avian influenza H5N1 virus [5].

However, unlike seasonal influenza in which around 90% of severe cases were the frail elderly, clinical data in the 2009 pandemic influenza showed that children and young adults under 60 accounted for about 68.7%-90% of the deaths (usually, in seasonal influenza, they account for about 17%) and 71% of the cases of severe pneumonia (usually, they account for 32%) [6,7]. The skewed age distribution toward children and young adults were similar to that in 2003 H5N1 epidemic, which had the mean age of 19.8 years [8], and similar to the 1918 H1N1 pandemic [9]. This age-related difference in infection and death rate could not be fully explained by social activity profiles as some household transmission studies clearly indicated that age is a protective factor [10,11]. Previous exposure to antigenically related influenza viruses was proposed as a mechanism of age protection [12,13], while lacking protective antibody and having immunocompromising underlying conditions have been proposed as two independent mechanisms of risk factors to infection and mortality from the 2009 H1N1 virus, because there are a percentage of death were apparently healthy young adults and children [14,15]. The age factor has not been addressed in animal models of 2009 H1N1 influenza pathogenesis and transmission. Animal age is either not specified [16] or only animals of one age, usually young adult, such as 13 month old ferret or 6-8 weeks mice [5,17,18], was used throughout a study.

In order to investigate the age factor for susceptibility and pathological response to 2009 H1N1 infection independent of preexisting cross-active antibodies in normal healthy individuals, we infected healthy influenza-naïve BALB/c mice of different ages with a 2009 A H1N1 influenza virus and compared their sensitivity to infection and pathology in lung and in spleen.

## Methods and materials

### Virus

A pandemic 2009 H1N1 virus, A/Beijing/501/09, was isolated from a confirmed H1N1 case in China. Virus was grown in the allantoic cavities of 10-day-old embryonated chicken eggs. Virus-containing allantoic fluid was harvested and stored in aliquots at -80°C until use. This virus does not carry the D222G mutation reported in some H1N1 pandemic strains. All infectious experiments were performed in an approved biosafety level 3 (BSL-3) facilities.

### Mice

Female BALB/c mice were provided by Laboratory Animal Center, Military Academy of Medical Sciences, Beijing, China. Mice were maintained in a specific pathogen-free facility and were housed in cages containing sterilized feed, autoclaved bedding and drinking water. The animal study protocol was approved by the Institutional Animal Care and Use Committee.

### LD_50 _of different mouse age groups

Female BALB/c mice, 4 week (young), 6 month (adult) and 1 year (older adult) of age, were anesthetized intraperitoneally (i.p.) with ketamine (75 mg/kg), and inoculated intranasally (i.n.) with A/Beijing/501/09 H1N1 virus at doses of 10^3^, 10^4 ^and 10^5 ^TCID_50 _(n = 10 for each age-dose group). In addition, a normal control group was set up for each age group which was given i.n. phosphate buffered saline (PBS). By 14 days after the infection, the end-point for the survival experiment, the survival rate was analyzed and lethal dose 50 (LD_50_) was calculated.

### Viral titers in tissues

Viral titers in lung and spleen were determined by TCID_50 _as described [19], except that TPCK-tripsin (Sigma-Aldrich, MO) was added into the medium (2 μg/ml) and 0.5% chicken erythrocytes were used in hemagglutination confirming cytopathic effect (CPE) endpoint.

### Immunohistochemistry

Immunohistochemical staining of H1N1 antigens in different group were performed using paraffin-embedded lung and spleen tissue. Sections of 4 μm thick were cut and mounted on charged glass slides. Sections were heated at 60°C for 1-2 hour and dewaxed in xylene, passed through graded alcohols, and rehydrated with water. Immunohistochemical staining was performed at room temperature. The reagent and wash buffer was 0.05 M Tris buffered saline plus 0.05% Tween 20 (TBST), pH7.6. Endogenous peroxidase activity was quenched by incubation in 3% hydrogen peroxide in TBST for ten minutes at room temperature. Slides were then washed and incubated for twenty minutes with 5% normal goat serum (Zhongshan Ltd, China) in TBST. Excess blocking serum was blown off, and the slides were incubated with polyclonal rabbit polyclonal anti-HA antibody of H1N1 (A/California/06/09) (Cat.IA-01SW-0100) (1:80) at 4°C overnight. Following washing, the slides were incubated for 1 hour in HRP/Fab polymer conjugated secondary antibody (Zhongshan Ltd, China), and visualized by incubation in diaminobenzidine (Zhongshan Ltd, China) for 5 minutes. The slides were counterstained for 10 seconds using hematoxylin (Zhongshan Ltd, China). Staining was negatively controlled by substituting rabbit immunoglobulin (Ig) fraction, diluted to the same Ig concentration, for the primary antibody. Stained sections were dehydrated and mounted.

The semiquantitative assessment of antigen expression on the 1 day after challenge in lungs including the bronchi and bronchioles was performed as reported [5].

### Histology

For pathologic analysis, three virus infected mice from each age group were euthanized at 1 d, 3 d and 5 d. The lungs and spleens were collected and fixed in 10% formalin at room temperature immediately and subsequently processed to paraffin embedding. Tissue sections of 4 μm from each animal were prepared and stained with hematoxylin and eosin for examination by light microscopy. All the samples were collected by a standardized protocol and not biased by changes seen in gross pathology. Parallel procedures were carried out for uninfected control mice for each age group.

Semi-quantitative assessment of influenza virus-associated inflammation and tissue damage in lungs and spleen were performed. The lung damage was evaluated as reported elsewhere [5] according to the degeneration and necrosis of bronchi and bronchiolar epithelium, the infiltration of inflammatory cells, alveoli degeneration and collapse, expansion of parenchymal wall and epithelial hemorrhage and edema. The cumulative scores of size and severity of degeneration or inflammation provided the total score per animal, and the average of three mice in a group was taken as the total score for that group.

The assessment of focal aggregation or induced bronchus-associated lymphoid node (iBALT) was performed by scoring the total number of iBALT on every slide, and the average score of three mice in a group was taken as the score of that group.

Semi-quantitative assessment of spleen damage was evaluated by the decrease of lymphocytes in splenic pulp and red pulp, the hyperemia and haemorrhage: 0 for normal spleen tissue, 1 for mild hyperemia and haemorrhage, 2 for moderate haemorrhage, decreased lymphocytes and increased phagocytes, 3 for no obvious spleen structure with severe decreased lymphocytes and more increased phagocytes.

All the evaluation of tissue damage was conducted by two independent observers. The average score of three mice in a group was taken as the score for that group.

### Statistical analysis

The significance between survival curves was analyzed by Kaplan-Meier survival analysis with log-rank test. Other data were analyzed using the 2-tailed Student's t test. *P *< 0.05 was considered significant. All analyses were performed with Graphpad Prism software.

## Results

### LD_50 _of different ages of mice infected with a 2009 H1N1 virus

To identify the susceptibility of different age of mice to 2009 H1N1 infection, different age group of mice were infected with different TCID_50 _virus. Data showed that the survival by 14 days after challenge was 0% for all mice in the 10^5 ^TCID_50 _groups; was 20%, 60%, 100% in the 10^4 ^TCID_50 _groups and 60%, 100%, 100% in the 10^3 ^TCID_50 _groups for the 4-week, 6-month and 1-year old mice, respectively. The 50% lethal dose (LD_50_) was calculated to be 10^3.3^, 10^4.2^, and 10^4.5 ^TCID_50 _for the 4-week, 6-month and 1-year old female BALB/c mice, respectively (Table 1). The results indicated that age is protective for pandemic H1N1 infection which parallels the human data.

**Table 1:** Survival of mice infected with a 2009 H1N1 influenza virus.

	Survival at 14 days after challenge (alive/total)			LD_50_
**Age\challenge dose**	**10**^**3**^**TCID**_**50**_	**10**^**4**^**TCID**_**50**_	**10**^**5**^**TCID**_**50**_	

4-week	6/10	2/10	0/10	10^3.3^TCID_50_

6-month	10/10	6/10	0/10	10^4.2^TCID_50_

1-year	10/10	10/10	0/10	10^4.5^TCID_50_

### Viral titers in tissues

**Figure 1:**
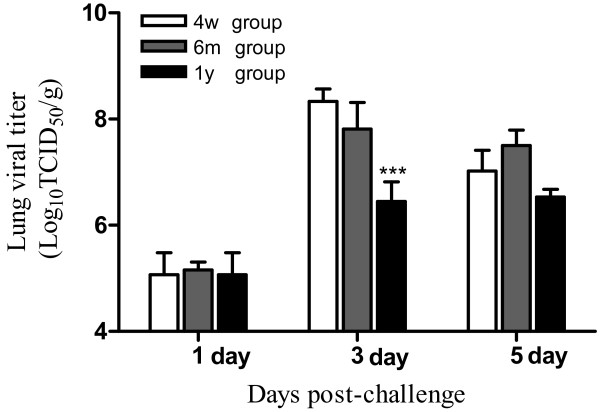
**Lung virus titers at 1, 3, and 5 days after H1N1 virus inoculation**. Female BALB/c mice, of 4-week, 6-month, and 1-year old, (n = 9 for each age group) were inoculated intranasally with a 10LD50 dose (10^4.3^, 10^5.2^, and 10^5.5 ^TCID_50_, respectively) dose of A/Beijing/501/09 H1N1 virus. At indicated time, the tissues were taken (n = 3 mice from each age group) and virus titer was determined. ***indicate *P *< 0.001 relative to the corresponding the different age group mice with 4 week age group mice. Mean ± SEM.

We further investigate the virus load and pathology of these three ages of mice infected by the A/Beijing/501/09 H1N1 virus at the dose of 10 LD_50 _for the respective age (Figure 1). At 1 day after infection, the three groups of mice had similar virus load in lung as justified by weight (Log_10_TCID_50_/g) and the numbers were close to the inoculation doses. At 3 days after, however, the lung viral titer in the 4-week and 6-month old mice were close to 3 log higher than those on day 1 while the viral titer in the 1-year old mice was less than 1.5 log higher than its level on day 1. By 5 days after infection, the lung viral titer in the 4-week group decreased over 1 log while the level in the two older groups remained relatively stable as compared to their day 3 level. No virus load was detected in the spleen of all age groups (Data not shown).

### Immunohistochemistry for H1N1 antigen distribution

**Figure 2:**
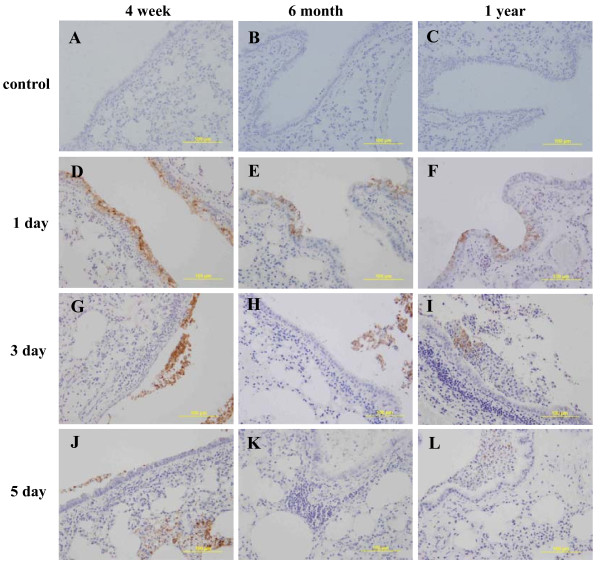
**Immunohistochemical staining of H1N1 antigen in mice lung**. The experiment set up is the same as in Figure 1 (inoculation at 10LD_50 _dose, n = 3 for each age group at each time point) except that there was a non-treated negative control group for each age. The tissues were stained with a polyclonal anti-HA antibody. No antigen was detected in the negative control group (A-C). At 1 day after inoculation, almost all the bronchi and bronchiolar epithelium cells in the 4-week age-group were positively stained (D), while less than half of the bronchi and bronchiolar epithelium cells were stained brown in the 6-month and 1-year age group (E,F). At 3 days and 5 days after inoculation, most positive stained bronchi and bronchiolar epithelium cells death and collapsed into bronchi and bronchiolar lumina (G-L). Magnification, × 200.

In order to identify if tissues from different ages of mice differ in their sensitivity to H1N1 infection, we performed immunohistochemical staining of mice lung and spleen taken from animals infected with 10 LD_50 _dose of 2009 H1N1 virus. Compared to the control group with no antigen detected in lung tissue (Figure 2A-C), in all three ages of mice, viral antigens were found in epithelial cells of bronchioles and terminal bronchioles, and alveolar macrophages, but not in alveolar epithelial cells. Significantly, at 1 day after infection, most of the epithelial cells of bronchus and bronchioles of the 4-week group mice were heavily stained while these cells in the 6-month and 1-year group mice were only partially stained and stained much lighter (Figure 2D-F) despite the inoculation of a higher infection dose for the older age groups (10^4.3^, 10^5.2^, and 10^5.5 ^TCID_50 _for the 4-week, 6-month and 1-year) respectively. At 3 days and 5 days after infection, lots of stained positive cells showed necrosis and detachment (Figure 2G-L). On the other hand, at all time points examined, there was no positive staining in spleen in all the age groups (data not shown). The results indicated different susceptibility of bronchiole epithelium cells to H1N1 virus exist in different group age mice.

### Lung damage in different age groups

**Figure 3:**
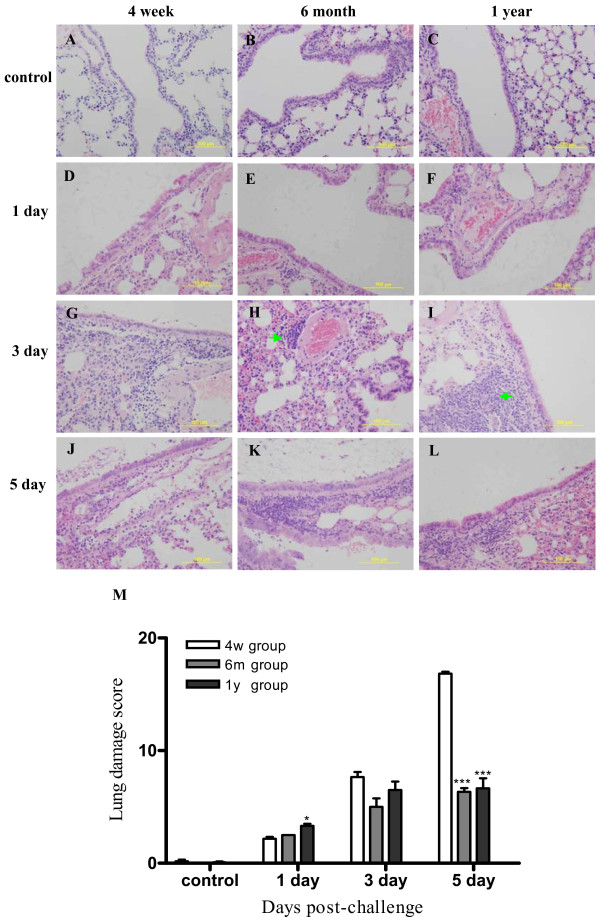
**Histopathologic development in mice inoculated with H1N1 virus**. The experiment set up is the same as in Figure 2. At 1 day after, the presentations were similar in all groups and there was little vacuolar degeneration of bronchi and bronchiole epithelium cells(D, E, F). At 3 days and 5 days after inoculation, the damage was severe in the 4-week group (G, J) than that of 6-month (H, K) and 1-year group (I, L). Severe interstitial edema was seen around blood vessels, apparent injured parenchyma and degenerated alveolar epithelial cells were seen with more inflammatory cells infiltration. The histological damage (M) was semi-quantified (n = 3 mce in each group). * and ***indicate *P *< 0.01 and *P *< 0.001 respectively relative to the corresponding the different age group mice with 4 week age group mice. Mean ± SEM. Magnification,× 200 (A-L).

**Figure 4:**
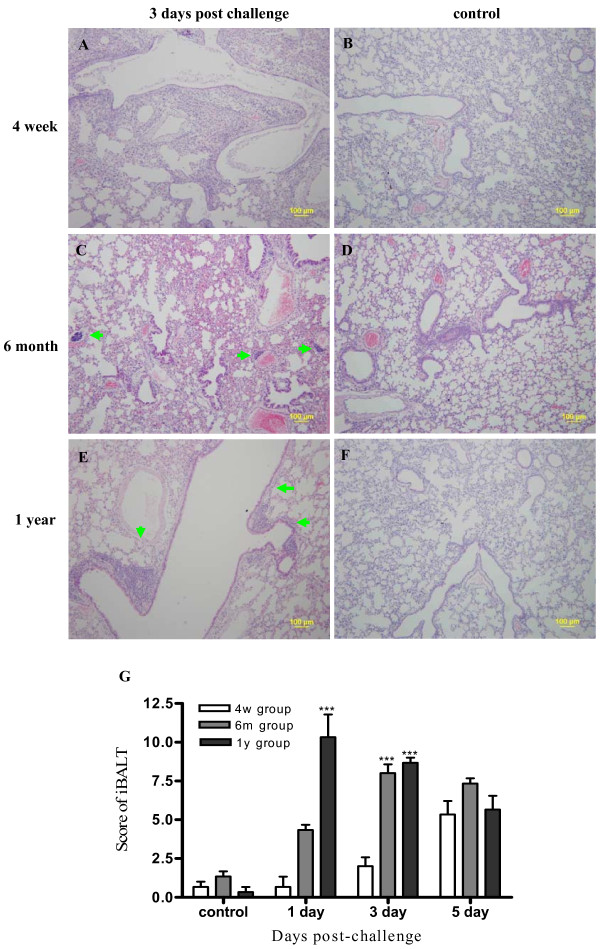
**Histopathologic examination of iBALT formation**. The experiment setup was the same as Figure 2 and histopathology was examined for day 1, 3 and 5 days after. The images of day 3 are shown (A-F) and semi-quantitation included all time points (G). (A-F) show the iBALT formation (light microscopy). Few iBALT was observed at 4 week group (A), while for the 6 month and 1 year groups, the number of iBALT (green arrow) especially those beside blood vessels or bronchials increased with age (C, E). No iBALT was observed in uninfected age control groups (B, D, F). Semi-quantification of iBALT formation (Figure G) indicated that iBALT formation, especially at three days after infection, increased with age. ***indicate *P *< 0.001 relative to the corresponding the different age group mice with 4 week age group mice. Mean ± SEM. (n = 3 mce in each group). Magnification, × 100 (A-F).

Compared to the control group (Figure 3A, B, C), the pathologic examination revealed that on day 1 after infection all three groups had similar presentations with vacuolar degeneration of bronchi and bronchiole epithelium cells, little increased broad interstitial and inflammatory cells with age (Figure 3 D, E, F). However on day 3 and 5 after infection the 4-week group mice presented much more severe lung damage than the two older groups especially the lung parenchyma. On day 3, in the 4-week group, the bronchi and bronchiolar epithelium of mice lung degenerated, showed multifocal necrosis and collapsed into bronchi and bronchiolar lumina with moderate numbers of lymphocytes or macrophages. The parenchyma around bronchial had apparent lesions and severe interstitial edema around blood vessels. Some alveolar epithelial cells denaturated and fell into alveolar lumina (Figure 3G). In the 6-month group, there were little lesions of alveolar epithelial cells, peribronchial and perivessle tissue. Some bronchus-associated lymphoid tissue (iBALT) developed beside blood vessels or bronchials (Figure 3H, Figure 4C). There was similar presentation of lung tissue in 1 year group (Figure 3I, 4E). No iBALT were observed in normal mice of each age group (Figure 4B, D, F). On day 5 after H1N1 inoculation, the difference of lung injury between the young group and the two adult groups was more distinguished with more extended degenerated and necrosis sites in the bronchiolar epitheliums, terminal bronchioles epitheliums, alveolar ducts and alveolar sacs and less recovered bronchi and bronchiolar epitheliums(Figure 3J). Besides, more edema and focal hemorrhage could be detected in 4-week group compared with 6 month and 1 year group (Figure 3K, L).

Semi-quantitative histological analysis indicated that the lung damage in young mice was more severe than that of adult aged mice after H1N1 inoculation (Figure 3M). In addition, semi-quantification analysis also indicated that iBALT formation, especially at the first three days after infection, increased with age (Figure 4G).

### Spleen damage in different age groups

**Figure 5:**
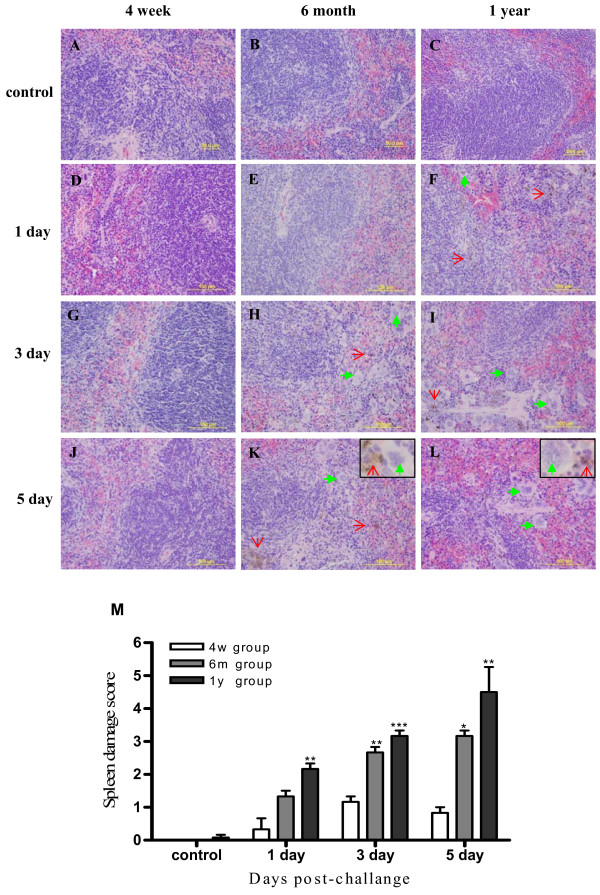
**Histopathological examination of spleens of mice infected with 2009 H1N1 virus**. The experiment set up is the same as in Figure 1. Compare to the control in different age groups (A-C), there was no significant difference at 1 day after except 1 year group with little increased phagocytes (D-F). At day 3 after H1N1 challenge, all three age groups presented hyperemia. The number of phagocytes (green arrow) in red pulp increased and lymphocytes in the white pulp decreased with age increased (G-I). At day 5 there was more severe hyperemia and focal haemorrhage than that at day 3 in each group. However, at 6 month group and 1 year group, hemosiderin (red arrow) could be easily seen in white pulp and red pulp with more severe decrease and apoptosis-like lymphocytes with age increased(J-L). (M) was the semi-quantitative histological analysis of spleen damage. *, ** and ***indicate *P *< 0.05, *P *< 0.01 and *P *< 0.001 respectively relative to the corresponding the different age group mice with 4 week age group mice. Mean ± SEM. n = 3 mice in each group. Magnification,× 400 (A-L).

To identify if different age group mice have distinguished immune response to H1N1 infection, the spleen pathological change were studied. Generally, on the first day after H1N1 challenge, the structure of spleens of the 4-week group mice was not different from that of control group (Figure 5A, D). However, the number of phagocytes in red pulp and artery cuff of spleen were relatively elevated in the 6-month and 1-year group (Figure 5E, F).

On day 3 after H1N1 challenge, the spleens of the 4-week group presented hyperemia with no obvious increase in phagocytes in red pulp as compared to day 1 (Figure 5G). However, in the 6-month group and 1-year group, the number of phagocytes in red pulp increased significantly (green arrow show), while lymphocytes in the white pulp did not decrease, and there was more severe hyperemia and focal haemorrhage than that on day 1. Besides hemosiderin (red arrow show) could be seen in white pulp and red pulp, which indicated haemorrhage and enhanced phagocytosis of macrophage (Figure 5H-I).

On day 5 after H1N1 challenge, although severe hyperemia presented in all groups, the number of phagocytes still did not show obvious increase in the 4-week group (Figure 5J), but the number of phagocytes increased dramatically in the 6-month and 1-year group with more hemosiderin detected (Figure 5K-L). Coincident with the enhanced phagocytosis, the number of spleen cells in adult mice especially in the 1-year group decreased dramatically.

Semi-quantitative histological analysis showed that spleen damage increased in severity with age, which perhaps had closed relations with the excessive activation of macrophage and then the decrease of lymphocytes in spleen (Figure 5M).

## Discussion

This study found that when challenged intranasally with the same dose of 2009 H1N1 virus, older mice are less likely to die than younger mice. This fact that age is protective for pandemic H1N1 infection parallels the human data. Importantly, in this mouse model of pandemic H1N1 infection, all mice had not been exposed to any influenza viruses before the experiment; therefore, pre-existing anti-H1N1 or cross-reactive anti-influenza immunity is not a mechanism to be considered for age-related protection. On the other hand, immune responses to pathogen infection differ by age [20-22], and age is a factor to be considered in vaccination and therapy strategies.

Mechanistic examination by immunohistochemistry and pathology revealed that young mice are more sensitive to 2009 influenza A H1N1 virus infection than older mice, at least partially due to the greater sensitivity of their bronchi epithelial cells. In the study by Munster et al. [16] using an adult ferret model, the 2009 H1N1 virus was found to replicate efficiently in the lower respiratory tract, and viral antigens were found in epithelial cells of bronchioles and terminal bronchioles, alveolar macrophages, and also type I and type II alveolar epithelial cells. In contrast, seasonal influenza virus was only found in the epithelial cells of the upper respiratory tract, and this difference in upper versus lower respiratory tract infection pattern was proposed as why the novel H1N1 virus will cause a larger epidemic than the seasonal influenza virus. This result is also in agreement with studies by Itho et al. and Belser et al. [18,23] that H1N1 viral antigens were detected in bronchiolar epithelia, desquamated cells in bronchiolar lumen, alveolar epithelium and histiocytes in thickening alveolus interstitial. In addition, our results from the murine model largely agree with the study in ferrets [16,17] in that we also found most virus infection in the lower respiratory tract and of the cell types infected, only marked difference was that mouse alveolar epithelial cells are not sensitive to H1N1 virus infection. This difference in cell type sensitivity might be due to animal species difference and might account for the relative insensitivity of mice as a species to influenza infection [24]. We suspect similar age-related sensitivity to H1N1 virus infection should also exist in ferrets and parallel study in ferrets should offer interesting insights to the age-distribution of the pandemic.

Related to immunohistochemical data, the virus lung titer assay affirmed more efficient virus proliferation in the younger mice in the first two to three days after infection. This higher virus titer might be explained by the more efficient infection at inoculation, as there will be more lung cells producing viruses, and it may or may not be due to more viruses made per mouse cell.

The iBALT, a tissue form that is not usually present in healthy animals [25,26] has been demonstrated to form upon certain infections and its formation is a protective immune response in that it clears influenza viral infection with a better organized immune response than that mediated by primary lymphoid organs which might over-react and result in more severe lung pathology and even death [27,28]. The role of innate response to influenza infection is complicated and can both help and inhibit viral clearance [29,30]. An effective level is necessary for infection clearance [31-33] but overreaction has been blamed to be causing deaths in young people in the H5N1 epidemic [34,35]. It has been reported that young mice have lower phagocytic functions and secret less TNF-alpha than adult mice [36,37], suggesting young mice has lower immunity than adult mice and has less ability to organize an efficient response for clearing influenza virus. Our data suggest that the moderate inflammatory response in lung of adult and old mice, organized with the help to iBALT, might provided an efficient level of protective immune response to the infection at earlier time after virus infection, while the young mice lacked iBALT formation and had insufficient level of inflammatory response for viral clearance and control.

H1N1 virus induces apoptosis of infected cells, which in a degree is a resistance mechanism of the body to viral replication [38]. In our study, at 3 days after virus infection, shedding of injured bronchial epithelial cells was observed, and some epithelium was seen in regeneration and repair stage. The regeneration of epithelium is slightly earlier in adult mice than in young mice (Figure 3D-F), suggesting adult mice have higher capacity in epithelium repair than young mice. The much more severe pathology observed in the lung of young mice as compared to adult and old mice appears to be explained by young mice's higher sensibility to infection, higher levels of virus replication and subsequent massive apoptosis and damage of their epithelium cells.

On the other hand, at the 10LD_50 _dose of H1N1 infection, we noted that the adult and older mice had much more severe damages in their spleen than the young mice, which might contribute to their eventual death. This data indicate that even the end result might be the same (although the older are more likely to survive a lower dose that's lethal to the young), young and older mice die from different pathological causes. If the same is true in humans, this serves a basis for adopting differential intervention strategies for different ages.

The murine influenza model has been widely used for studying influenza pathogenesis, viral therapy and vaccines [39] and the model has been used to study this 2009 H1N1 virus. Pathological findings in mice mimic that in the clinic [40]. In addition, mice are the animal of choice for studying immune responses at different ages and development stages [41-43]. The current study found that 1) young mice are more susceptible to the 2009 pandemic influenza A H1N1 virus infection and are more likely to die from the infection than adult and old mice, which parallels the pandemic epidemiology in humans; 2) this age-related sensitivity to H1N1 infection might be explained by young mice's higher sensitivity of its bronchial and bronchiole epithelial cells to be infected, a weaker innate immune response of the young mice to control and clear infection, and therefore young mice are more likely to die from infection-caused massive apoptosis and damage of mouse bronchial epithelial cells and the lung structure and function. This is a first age-comparison study in animals for 2009 H1N1 infection, and by using an influenza-naive and immunocompetent mouse model it studied the age factor independently of two major influential factors of the pandemic. The study highlights the importance of age factor in the 2009 H1N1 virus infection and also suggests that animal model data with unspecified age or one age should be interpreted with caution. Despite the imperfections of the animal model, these observations might provide some mechanistic explanations for the age distribution in the 2009 human influenza pandemic.

## Conclusions

The age factor should be considered when studying the pathogenesis and transmission of influenza virus and formulating the vaccination and therapy strategies. The present study explored that young individuals are more sensitive to H1N1 infection and have less protective immune responses than older adults.

## Competing interests

The authors declare that they have no competing interests.

## Authors' contributions

SS, GZ and YZ designed research. SS, GZ, WX, JH, YG, HY and XW performed research. SS, GZ and YT analyzed data. SS, GZ, YT and YZ wrote and modified the paper. SS and GZ have equal contributions to this paper. All authors read and approved the final manuscript.

## References

[B1] Garten RJ Davis CT Russell CA Shu B Lindstrom S Balish A Sessions WM Xu X Skepner E Deyde V Okomo-Adhiambo M Gubareva L Barnes J Smith CB Emery SL Hillman MJ Rivailler P Smagala J de Graaf M Burke DF Fouchier RA Pappas C Alpuche-Aranda CM Lopez-Gatell H Olivera H Lopez I Myers CA Faix D Blair PJ Yu C Antigenic and genetic characteristics of swine-origin 2009 A(H1N1) influenza viruses circulating in humans Science 2009 325 197 201 10.1126/science.1176225 19465683 PMC3250984

[B2] Statement to the press by WHO Director-General Dr Margaret Chan: World now at the start of 2009 influenza pandemic http://www.who.int/mediacentre/news/statements/2009/h1n1_pandemic_phase6_20090611/en/

[B3] Pandemic (H1N1) 2009 - update 93 http://www.who.int/csr/disease/swineflu/laboratory26_03_2010/en/

[B4] Jain R Goldman RD Novel influenza A(H1N1): clinical presentation, diagnosis, and management Pediatr Emerg Care 2009 25 791 796 10.1097/PEC.0b013e3181c3c8f8 19915434

[B5] van den Brand JM Stittelaar KJ van Amerongen G Rimmelzwaan GF Simon J de Wit E Munster V Bestebroer T Fouchier RA Kuiken T Osterhaus AD Severity of pneumonia due to new H1N1 influenza virus in ferrets is intermediate between that due to seasonal H1N1 virus and highly pathogenic avian influenza H5N1 virus J Infect Dis 2010 201 993 999 10.1086/651132 20187747 PMC7110095

[B6] Gordon SM Update on 2009 pandemic influenza A (H1N1) virus Cleve Clin J Med 2009 76 577 582 10.3949/ccjm.76a.05009 19797457

[B7] Influenza and Pandemic H1N1 2009 Bulletin http://www.wpro.who.int/NR/rdonlyres/B89007E1-FC03-4060-848C-E3616E333B3B/0/H1N1BulletinVol2Issue120100119.pdf

[B8] Smallman-Raynor M Cliff AD Avian influenza A (H5N1) age distribution in humans Emerg Infect Dis 2007 13 510 512 10.3201/eid1303.060849 17552119 PMC2141519

[B9] Azambuja MI A parsimonious hypothesis to the cause of influenza lethality and its variations in 1918-1919 and 2009 Med Hypotheses 74 81 684 19962834 10.1016/j.mehy.2009.10.050 PMC7130991

[B10] auchemez S Donnelly CA Reed C Ghani AC Fraser C Kent CK Finelli L Ferguson NM Household transmission of 2009 pandemic influenza A (H1N1) virus in the United States N Engl J Med 2009 361 2619 2627 10.1056/NEJMoa0905498 20042753 PMC3840270

[B11] France AM Jackson M Schrag S Lynch M Zimmerman C Biggerstaff M Hadler J Household transmission of 2009 influenza A (H1N1) virus after a school-based outbreak in New York City, April-May 2009 J Infect Dis 201 984 992 10.1086/651145 20187740

[B12] Miller E Hoschler K Hardelid P Stanford E Andrews N Zambon M Incidence of 2009 pandemic influenza A H1N1 infection in England: a cross-sectional serological study Lancet 375 1100 1108 10.1016/S0140-6736(09)62126-7 20096450

[B13] Miller M Viboud C Simonsen L Olson DR Russell C Mortality and morbidity burden associated with A/H1N1pdm influenza virus PLoS Curr 2009 1 RRN1013 10.1371/currents.RRN1013 20029607 PMC2762375

[B14] Bettinger JA Sauve LJ Scheifele DW Moore D Vaudry W Tran D Halperin SA Pelletier L Pandemic influenza in Canadian children: a summary of hospitalized pediatric cases Vaccine 28 3180 3184 10.1016/j.vaccine.2010.02.044 20189488

[B15] Who is more at risk of severe illness? What about other risks? http://www.who.int/csr/disease/swineflu/frequently_asked_questions/risk/en/

[B16] Munster VJ de Wit E van den Brand JM Herfst S Schrauwen EJ Bestebroer TM van de Vijver D Boucher CA Koopmans M Rimmelzwaan GF Kuiken T Osterhaus AD Fouchier RA Pathogenesis and transmission of swine-origin 2009 A(H1N1) influenza virus in ferrets Science 2009 325 481 483 19574348 10.1126/science.1177127 PMC4814155

[B17] Maines TR Jayaraman A Belser JA Wadford DA Pappas C Zeng H Gustin KM Pearce MB Viswanathan K Shriver ZH Raman R Cox NJ Sasisekharan R Katz JM Tumpey TM Transmission and pathogenesis of swine-origin 2009 A(H1N1) influenza viruses in ferrets and mice Science 2009 325 484 487 19574347 10.1126/science.1177238 PMC2953552

[B18] Itoh Y Shinya K Kiso M Watanabe T Sakoda Y Hatta M Muramoto Y Tamura D Sakai-Tagawa Y Noda T Sakabe S Imai M Hatta Y Watanabe S Li C Yamada S Fujii K Murakami S Imai H Kakugawa S Ito M Takano R Iwatsuki-Horimoto K Shimojima M Horimoto T Goto H Takahashi K Makino A Ishigaki H Nakayama M In vitro and in vivo characterization of new swine-origin H1N1 influenza viruses Nature 2009 460 1021 1025 19672242 10.1038/nature08260 PMC2748827

[B19] Zhao G Lin Y Du L Guan J Sun S Sui H Kou Z Chan CC Guo Y Jiang S Zheng BJ Zhou Y An M2e-based multiple antigenic peptide vaccine protects mice from lethal challenge with divergent H5N1 influenza viruses Virol J 2010 7 9 10.1186/1743-422X-7-9 20082709 PMC2823673

[B20] Liang S Domon H Hosur KB Wang M Hajishengallis G Age-related alterations in innate immune receptor expression and ability of macrophages to respond to pathogen challenge in vitro Mech Ageing Dev 2009 130 538 546 10.1016/j.mad.2009.06.006 19559723 PMC2717634

[B21] Maue AC Yager EJ Swain SL Woodland DL Blackman MA Haynes L T-cell immunosenescence: lessons learned from mouse models of aging Trends Immunol 2009 30 301 305 10.1016/j.it.2009.04.007 19541537 PMC3755270

[B22] Zaghouani H Hoeman CM Adkins B Neonatal immunity: faulty T-helpers and the shortcomings of dendritic cells Trends Immunol 2009 30 585 591 10.1016/j.it.2009.09.002 19846341 PMC2787701

[B23] Belser JA Wadford DA Pappas C Gustin KM Maines TR Pearce MB Zeng H Swayne DE Pantin-Jackwood M Katz JM Tumpey TM Pathogenesis of pandemic influenza A (H1N1) and triple-reassortant swine influenza A (H1) viruses in mice J Virol 2010 84 4194 4203 10.1128/JVI.02742-09 20181710 PMC2863721

[B24] van Riel D Munster VJ de Wit E Rimmelzwaan GF Fouchier RA Osterhaus AD Kuiken T Human and avian influenza viruses target different cells in the lower respiratory tract of humans and other mammals Am J Pathol 2007 171 1215 1223 10.2353/ajpath.2007.070248 17717141 PMC1988871

[B25] Lipscomb MF Hutt J Lovchik J Wu T Lyons CR The pathogenesis of acute pulmonary viral and bacterial infections: investigations in animal models Annu Rev Pathol 5 223 252 10.1146/annurev-pathol-121808-102153 19824827

[B26] Tschernig T Pabst R Bronchus-associated lymphoid tissue (BALT) is not present in the normal adult lung but in different diseases Pathobiology 2000 68 1 8 10.1159/000028109 10859525

[B27] Hui KP Lee SM Cheung CY Ng IH Poon LL Guan Y Ip NY Lau AS Peiris JS Induction of proinflammatory cytokines in primary human macrophages by influenza A virus (H5N1) is selectively regulated by IFN regulatory factor 3 and p38 MAPK J Immunol 2009 182 1088 1098 19124752 10.4049/jimmunol.182.2.1088

[B28] Moyron-Quiroz JE Rangel-Moreno J Kusser K Hartson L Sprague F Goodrich S Woodland DL Lund FE Randall TD Role of inducible bronchus associated lymphoid tissue (iBALT) in respiratory immunity Nat Med 2004 10 927 934 10.1038/nm1091 15311275

[B29] Kuiken T Taubenberger JK Pathology of human influenza revisited Vaccine 2008 26 Suppl 4 D59 66 10.1016/j.vaccine.2008.07.025 19230162 PMC2605683

[B30] Peiris JS Cheung CY Leung CY Nicholls JM Innate immune responses to influenza A H5N1: friend or foe? Trends Immunol 2009 30 574 584 10.1016/j.it.2009.09.004 19864182 PMC5068224

[B31] Kolls JK McCray PB Jr Chan YR Cytokine-mediated regulation of antimicrobial proteins Nat Rev Immunol 2008 8 829 835 10.1038/nri2433 18949018 PMC2901862

[B32] Rangel-Moreno J Moyron-Quiroz JE Hartson L Kusser K Randall TD Pulmonary expression of CXC chemokine ligand 13, CC chemokine ligand 19, and CC chemokine ligand 21 is essential for local immunity to influenza Proc Natl Acad Sci USA 2007 104 10577 10582 10.1073/pnas.0700591104 17563386 PMC1965555

[B33] Medzhitov R Origin and physiological roles of inflammation Nature 2008 454 428 435 10.1038/nature07201 18650913

[B34] Lee SM Gardy JL Cheung CY Cheung TK Hui KP Ip NY Guan Y Hancock RE Peiris JS Systems-level comparison of host-responses elicited by avian H5N1 and seasonal H1N1 influenza viruses in primary human macrophages PLoS One 2009 4 e8072 10.1371/journal.pone.0008072 20011590 PMC2788213

[B35] Perrone LA Plowden JK Garcia-Sastre A Katz JM Tumpey TM H5N1 and 1918 pandemic influenza virus infection results in early and excessive infiltration of macrophages and neutrophils in the lungs of mice PLoS Pathog 2008 4 e1000115 10.1371/journal.ppat.1000115 18670648 PMC2483250

[B36] Kethineni N Brummer E Stevens DA Susceptibility to pulmonary blastomycosis in young compared to adult mice: immune deficiencies in young mice Med Mycol 2006 44 51 60 10.1080/13693780500220498 16805093

[B37] Zhou J Law HK Cheung CY Ng IH Peiris JS Lau YL Functional tumor necrosis factor-related apoptosis-inducing ligand production by avian influenza virus-infected macrophages J Infect Dis 2006 193 945 953 10.1086/500954 16518756 PMC7109654

[B38] Mok CK Lee DC Cheung CY Peiris M Lau AS Differential onset of apoptosis in influenza A virus H5N1- and H1N1-infected human blood macrophages J Gen Virol 2007 88 1275 1280 10.1099/vir.0.82423-0 17374772

[B39] Mizgerd JP Skerrett SJ Animal models of human pneumonia Am J Physiol Lung Cell Mol Physiol 2008 294 L387 398 10.1152/ajplung.00330.2007 18162603

[B40] Perez-Padilla R de la Rosa-Zamboni D Ponce de Leon S Hernandez M Quinones-Falconi F Bautista E Ramirez-Venegas A Rojas-Serrano J Ormsby CE Corrales A Higuera A Mondragon E Cordova-Villalobos JA Pneumonia and respiratory failure from swine-origin influenza A (H1N1) in Mexico N Engl J Med 2009 361 680 689 10.1056/NEJMoa0904252 19564631

[B41] Barrios C Brawand P Berney M Brandt C Lambert PH Siegrist CA Neonatal and early life immune responses to various forms of vaccine antigens qualitatively differ from adult responses: predominance of a Th2-biased pattern which persists after adult boosting Eur J Immunol 1996 26 1489 1496 10.1002/eji.1830260713 8766551

[B42] Po JL Gardner EM Anaraki F Katsikis PD Murasko DM Age-associated decrease in virus-specific CD8+ T lymphocytes during primary influenza infection Mech Ageing Dev 2002 123 1167 1181 10.1016/S0047-6374(02)00010-6 12044966

[B43] Toapanta FR Ross TM Impaired immune responses in the lungs of aged mice following influenza infection Respir Res 2009 10 112 10.1186/1465-9921-10-112 19922665 PMC2785782

